# The Impact of the 2008 Economic Crisis on Substance Use Patterns in the Countries of the European Union

**DOI:** 10.3390/ijerph13010122

**Published:** 2016-01-13

**Authors:** Geert Dom, Jerzy Samochowiec, Sara Evans-Lacko, Kristian Wahlbeck, Guido Van Hal, David McDaid

**Affiliations:** 1 Collaborative Antwerp Psychiatric Research Institute (CAPRI), Antwerp University, Antwerp 2640, Belgium; 2 Department of Psychiatry, Pomeranian Medical University, Szczecin 71-460, Poland; samoj@pum.edu.pl; 3 Health Service and Population Research Department, Institute of Psychiatry, King’s College London, SE5 8AF, UK; sara.evans-lacko@kcl.ac.uk; 4 The Finnish Association for Mental Health, Helsinki 00240, Finland; Kristian.Wahlbeck@mielenterveysseura.fi; 5 Medical Sociology and Health Policy, Antwerp University, Antwerp 2640, Belgium; guido.vanhal@uantwerpen.be; 6 Personal Social Services Research Unit, London School of Economics and Political Science, London WC2A 2AE, UK; D.Mcdaid@lse.ac.uk

**Keywords:** economic crisis, European Union, substance use, alcohol, drugs, nicotine

## Abstract

*Background*: From 2008 on, a severe economic crisis (EC) has characterized the European Union (E.U.). However, changes in substance use behavioral patterns as a result of the economic crisis in Europe, have been poorly reflected upon, and underlying mechanisms remain to be identified; *Methods*: In this review we explore and systematize the available data on the effect of the 2008 economic crisis on patterns of substance use and related disorders, within the E.U. countries; *Results*: The results show that effects of the recession need to be differentiated. A number of studies point to reductions in population’s overall substance use. In contrast, an increase in harmful use and negative effects is found within specific subgroups within the society. Risk factors include job-loss and long-term unemployment, and pre-existing vulnerabilities. Finally, our findings point to differences between types of substances in their response on economic crisis periods; *Conclusions*: the effects of the 2008 economic crisis on substance use patterns within countries of the European Union are two-sided. Next to a reduction in a population’s overall substance use, a number of vulnerable subgroups experience serious negative effects. These groups are in need of specific attention and support, given that there is a real risk that they will continue to suffer negative health effects long after the economic downfall has formally been ended.

## 1. Introduction

Since 2008, a severe economic crisis (EC) has characterized the European Union (E.U.). The countries most severely impacted were those countries whose banking systems have been most exposed to the economic crisis; *i.e.*, Cyprus, Greece, Ireland, Italy, Portugal and Spain [1]. However, there is growing evidence that the effects are seen well beyond these countries impacting a broad set of social, economic and health domains [2]. It is within this context that the 2010 European Monitoring Centre for Drugs and Drug Addiction (EMCDDA) Annual Report noted that economic slowdown has produced “fears that this may be accompanied by an increase in problematic forms of drug use”.

However, until now a systematic overview of the E.U. data that substantiate these fears has not been done. Indeed, although a sizeable number of studies have explored the relationship between (earlier) economic crises and changes in substance use patterns, throughout many countries in the world, the European context has been as yet been poorly explored [3]. An additional complicating factor is that the E.U. context is characterized by great differences between countries in a number of dimensions, *i.e.*, the impact of the crisis, traditional substance use patterns, and legal regulation systems.

In addition to systematize epidemiological data there is a strong need to find explanatory mechanisms that mediate the relation between EC and changes in substance use patterns within a population. Identifying specific mechanisms is important in developing more targeted interventions to decrease the effect of EC on substance use. Based upon the findings in their systematic review, including studies worldwide, de Goeij *et al.* suggest two mechanisms. First, the psychological distress triggered by EC’s consequences explains an increase of harmful substance use, specifically in those subgroups most affected by the EC. The second mechanism, suggests that due to tighter budget constraints, less money is spent on psychoactive substances such as alcohol or nicotine [3]. However, many other mechanisms may play a role [4,5].

Taken together, changes in substance use behavioral patterns as a result of the economic crisis in Europe, which started in 2008, have been poorly reflected upon, and underlying mechanisms remain to be identified. This represents a serious caveat, especially within the context of the current ongoing processes in which the E.U. is developing joint action plans and policies on alcohol, tobacco and drug-related issues as well as on broader mental health domains.

Therefore, the aim of the current review is to explore the relationship between the “Great Recession” and changes in patterns of substance use and related disorders, within the European Union countries. In addition we explored whether the E.U. data give support to the dual effects of the EC, *i.e.*, reduction of substance use within the general population and increase of harmful substance use patterns within vulnerable subgroups, as suggested by de Goeij *et al.* [3].

## 2. Method

We used the Pubmed database with the search terms (alcohol or drugs or nicotine or smoking) or (alcohol or drugs or nicotine) abuse or dependence and (economic crisis or business cycles) AND Europe, within the title or abstract. Limitations were set on studies published from 1 January 2008 to 31 May 2015. This search procured 75 hits. 

**Figure 1:**
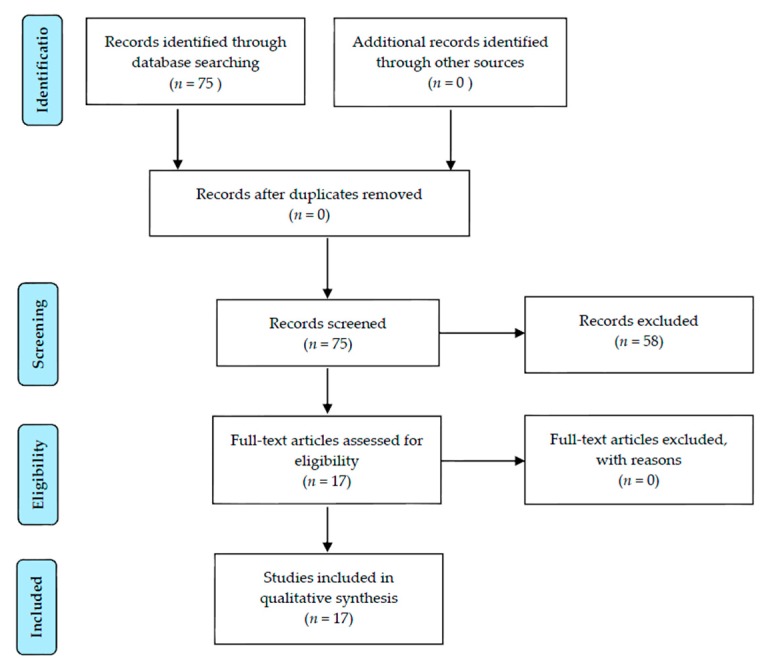
Prisma flow diagram.

All abstracts of the articles were screened (by first author Geert Dom) to identify literature relating to our specific focus: the European Union, the 2008 economic crisis and its aftermath in relation to changes in alcohol, drug, or smoking behaviors within the general population. Review articles (*n* = 8) and articles not having as primary focus data on alcohol, illicit drug, or smoking behaviors in relation to economic crisis were excluded (*n* = 50). We limited our search to original research studies providing data in countries belonging to the European Union and candidate countries. References of the selected articles were screened for additional literature (*n* = 0). The corresponding Prisma flowchart is shown in Figure 1.

## 3. Results

Ultimately 17 original research articles were included, 11 on alcohol, three on drugs, and three on cigarette smoking.

### 3.1. Alcohol

We found eleven studies on alcohol and related disorders (Table 1). Two show a decrease in the general population’s alcohol consumption and related harm during EC periods. Six studies report negative effects of economic crisis periods on alcohol consumption, most frequently associated with unemployment status. Three studies showed mixed results, *i.e.*, a reduction of heavy drinking patterns but increases in binge drinking.

In Italy, although all-cause mortality remained stable during economic fluctuations, alcohol consumption increased in 2009, the year with the worst real GDP decrease (−5.1%) [6]. In England, comparing data between 2004 and 2010, there was a remarkable decrease in frequent (hazardous) drinking following the onset of the banking crisis in 2008/2009 [7]. However, in this study there was a notable rise of binge drinking among a smaller high-risk group of unemployed drinkers [7]. These findings are in line with a recent Spanish study showing that during the period of economic recession in Spain heavy drinking decreased and binge drinking increased [8]. In another, Spanish study comparing the pre- and post-crisis years 2006 and 2010, a significant rise in alcohol dependence and related disorders was found, specifically among those who experienced severe economic losses, e.g., unemployment [9].

In the Nordic countries such as Sweden a deep recession occurred earlier (1992–1996). Some of the adverse effects of the recession persisted long after the crisis had ended. Between 1997 and 2002 there was an increase in the hazard of alcohol disease-related mortality, in line with the duration of unemployment for women, while for men mortality peaked with middle-level of unemployment duration [10]. The importance of job-loss was underlined in another longitudinal Swedish study, where job-loss was associated with an excess risk of both alcohol-related hospitalization and mortality among both displaced men and women. For women, alcohol-attributable health problems were mainly limited to alcohol use disorders, whereas men also had an increased risk of hospitalization from poisoning and alcohol-induced liver disease and pancreatitis [11]. In Finland, a worsening alcohol-related mortality was associated with improvement in macro-economic conditions [12,13].

**Table 1:** Studies on alcohol and alcohol use disorders during and after the economic crisis in European Union countries.

Author [Reference]	Sample	Period Survey	Country	Outcome
Mattei *et al.*, 2014 [6]	Data from Italian government agencies	2000 *vs.* 2010	Italy	Increased population alcohol consumption in 2009.
Harray *et al.*, 2014 [7]	Nationally representative sample of non-institutionalized white persons aged 20–60 years from seven waves of the Health Survey for England, 2004–2010 (*n* = 36,525)	2004 *vs.* 2010	UK	Decreased frequent (hazardous) drinking in 2008/2009 and rise of binge drinking among unemployed drinkers.
Gili *et al.*, 2013 [9]	Samples of patients attending primary care centers representing Spain's consulting populations (*n* = 7940)	2006/2007 *vs.* 2010/2011	Spain	4.6% rise of alcohol dependence and related disorders, specifically within those with severe economic losses
Garcy and Vagero, 2012 [10]	All Swedish men and women (*n* = 3,392,169) born between 1931 and 1965, and who were still alive on 31 December 1996. (Data: The Work and Mortality Database, WMD)	1997 *vs.* 2002	Sweden	Increased hazard alcohol-related mortality associated with duration unemployment
Eliason *et al.*, 2014 [11]	Employee-employer data merged with information on alcohol-attributable deaths and hospital admissions from the Causes of Death Register and the National Patient Register	Employee-employer data were used to identify all establishment closures during 1990–1999. Follow-up for 12 years	Sweden	Job-loss associated with increased alcohol-related mortality and morbidity
Herttua *et al.*, 2007 [12]	A register-based follow-up study of all over 15-year-old Finnish men and women.	1975 *vs.* 2001	Finland	Improvement of macroeconomic conditions associated with decreased alcohol-related mortality
Johansson *et al.*, 2006 [13]	Cross-sectional population survey “health 2000 in Finland“ (*n* = 10,000) carried out in 2000	1975 *vs.* 2001	Finland	Decreased alcohol-related mortality in the early 1990’s
Stuckler *et al.*, 2009 [14]	Age-standardized and age-specific mortality data from the WHO European Health for All Database	1970–2007	26 European countries	Rise of unemployment associated with excess alcohol-related mortality
Toffolutti and Suhrcke, 2014 [15]	Unemployment rate as the main indicator for the macroeconomic fluctuation. Health indicator the overall mortality rate, selected cause-specific mortality rates, as well as health behavior proxies.	2003 *vs.* 2010	23 European countries	Short-term effects crisis; decreased total rate alcohol consumption and mortality due to liver disease
Colell *et al.*, 2015 [8]	Cross-sectional data from four editions of the Spanish Household Survey on Alcohol and Drugs, economically active individuals aged 16–64 years (total sample = 62,440).	2005/2007 (P1) *vs.* 2009/2011 (P2)	Spain	During P2: Heavy drinking (daily average >40 g. for men and 24 g. for women) decreased for both men and women. Increased binge drinking (>5 units/2 h) in both men and women

Overall there seems to be an association between alcohol use related morbidity and unemployment. In a large study in 26 European countries, a 3% increase in unemployment was associated with excess deaths from alcohol abuse during the economic downfall (excess deaths E.U.-wide) [14].

This is consistent with a recent study on the short-term health effects of the economic crisis using data from 23 European countries, which reported an overall decrease in the total rate of alcohol consumption and mortality due to chronic liver diseases [15].

### 3.2. Smoking

Only three studies on smoking and economic crisis could be found (Table 2). In Italy, comparing 2009 (the height of the Italian crisis) with 2008 data, a significant increase of smoking prevalence in both women and men was reported. Of interest, there was no change in the number of never smokers in this study, leading the authors to believe that former smokers relapsed in their old smoking patterns [16]. 

**Table 2:** Studies on cigarette smoking during and after the economic crisis in European Union countries.

Author [Reference]	Sample	Period Survey	Country	Outcome
Gallus *et al.*, 2011 [16]	Representative general population survey (*n_(2008)_* = 3085, *n_(2009)_* = 3213)	2008 *vs.* 2009	Italy	Compared to 2008 significantly increased smoking prevalence in 2009; 22.0% to 25.4%, in both men and women. Number of never smokers remained the same
Tarantilis *et al.*, 2015 [17]	Econometric modeling of cigarette consumption with different cigarette price and gross domestic product fluctuation scenarios	1994–2012	Greece	Decreased cigarette consumption
Schoretsaniti *et al.*, 2014 [18]	Trend analysis of three representative national and cross-sectional surveys	2006–2010, 2011	Greece	Increased number people with intention to quit smoking. Quit attempts more frequent in people with high socioeconomic status.

Although Tarantelli *et al.* [17] report in their study a sharp drop in overall cigarette consumption during the last decades, this study does not provide original data but rather gives an econometric estimation of changes of cigarette consumption in function of changes in taxation rates and incomes. Also in Greece, and in part probably due to a combination of tobacco control policies and austerity measures, the intention to quit smoking has increased, although actual quit attempts were higher among those less disadvantaged potentially widening inequalities between socio-economic groups [18]. Taken together, only one study provides data on changes in smoking prevalence’s [16], with two studies providing information on smoking proxy-variables [17,18].

### 3.3. Illicit Drugs

Although it might be expected that any economic downturn could potentially have a great effect on changes in drug use patterns, the number of studies within the European context is remarkably limited (Table 3). Between 2008 and 2010 there was a substantial increase in the number of persons with problematic drug use in Greece. The increase was most notable among older persons [19]. Taking into account that in 2010 the average age in Greece for initiation of injection drug use was estimated at 22.4 years old the authors suggest that the sharp increase of older heroin users in Greece indicates probable relapses rather than new cases, presumably associated with the ongoing economic downturn. Analysis of wastewater in two North-Italian cities showed a decrease in use of more expensive drugs (*i.e.*, heroin and cocaine) and an increase in cheaper drugs (cannabis and amphetamine) [20]. In Spain a recent study reported that sporadic cannabis use increased among older unemployed men and women [8].

**Table 3:** Studies on drug use and drug use disorders during and after the economic crisis in European Union countries.

Author [Reference]	Sample	Period Survey	Country	Outcome
Zuccato *et al.*, 2012 [20]	Urban wastewater analysis in two cities	2005 *vs.* 2009	Italy Wastewater analysis in two cities	Decrease of expensive drugs and increase cheap drugs in wastewater analyses.
Kondilis *et al.*, 2013 [19]	Based upon national statistic health data	Data EMCDDA 2008 *vs.* 2010	Greece	Number of persons with problematic drug use rose by 11.6% between 2008 and 2010. Among those aged 35 to 64 years, the increase was far more intense at 88.2%.
Colell *et al.*, 2015 [8]	Data from the Spanish Household Survey on Alcohol and Drugs (EDADES). Representative population survey age 15–64 (*n* = 62,440)	2005/2007 (P1) *vs.* 2009/2011(P2)	Spain	Unemployed men and women increased sporadic use cannabis *vs.* employed.

## 4. Discussion

Although substance use and related disorders are a major public health problem only a surprisingly low number of studies explored the impact of the recent economic crisis on substance use patterns in E.U. countries. In addition the wide variability in the used methodology and sample choices provide mostly fragmented information on the topic chosen and make interpretation of results difficult.

Most studies focused on alcohol. The results show that effects of the recession need to be differentiated, and give some support for a bidirectional effect. On the one hand a number of studies point to reductions in population’s overall substance use. On the other hand an increase in harmful use and negative effects is found within specific subgroups within the society. Risk factors include job-loss and long-term unemployment, and pre-existing vulnerabilities. Finally, our findings point to differences between types of substances in their response on economic crisis periods.

Overall a majority of the studies included in this review provide indications that within different European countries the economic crisis has been paralleled with a reduction of use and heavy use in the general population. This accounts specifically for drinking alcohol and cigarette smoking but interestingly not for illicit drugs. These findings are consistent with findings in other non-E.U. countries and different crisis time periods. The EC in Iceland led to large and significant reductions in health behaviors such as alcohol consumption and cigarette smoking [21]. In the U.S. there was a tendency during economic crisis periods for a large part of the population to buy and use less alcoholic beverages [22,23]. This was reflected in market reductions and decreases in hazardous drinking and drink-driving prevalence (although this latter relationship may be biased by an overall reduction in car-possession and driving due to economic hard-ships) [7,24]. The findings are also consistent with a Canadian study comparing data between 2003 and 2010. Canada experienced substantial negative economic growth in 2009 and a one-point increase in the unemployment rate was associated with 0.15% fewer drinks consumed in the past month and a 0.14% decrease in past-month heavy drinking, specifically in men [25].

Taken together, economic crisis periods seem to have a down regulatory effect on the use of alcohol and tobacco in the population, which affect the populations health positively. Price regulation and budget restriction are probably the main driving mechanisms [26]. However, we need to take into account that the number of studies in support of these hypotheses remains low, so studies in more (European) countries with different social systems are clearly needed.

In contrast to these general population’s positive effects the findings in this overview show that specific subgroups are very negatively affected by the EC. In current review this was most consistently documented for alcohol use in association with people suffering from job-loss or unemployment [7,9,10,11,14,27], and to a lesser degree regarding tobacco [18], and illicit drug use [8]. The findings on the alcohol-unemployment association were very recently confirmed in a multi-country European study published after closing time of our review [28].

Most studies in this overview highlight the negative effect of job-loss and unemployment on substance use patterns; more risky use (binge drinking), alcohol-related hospitalization and mortality, and illicit drug use. The increase in binge-drinking patterns that is reported in many studies is important. Indeed, binge-drinking patterns have been associated with a higher risk on medical, mental health, and social adversities [29,30].

Overall, these findings are consistent with other non-E.U. countries reporting increases in alcohol use, alcohol use disorders, and alcohol-related morbidity and mortality, associated with unemployment within a substantial part of the population [14,22,31,32,33].

The economic crisis has affected employment rates on a major scale. Stuckler *et al.* [34] found that in 26 European countries, job loss increased above the 2007 level after 2009. Unemployment appears to be a major driver of increased mental health problems, both during “economic normal” and economic crisis periods. Some authors have suggested a dose-response effect [24]. Our findings on substance use patterns point in the same dose-response direction. In the largest study included in the review, over 26 European countries, a 3% increase in unemployment was associated with excess deaths from alcohol abuse during the economic downfall (excess deaths E.U.-wide) [14]. Of importance, not only the population prevalence but also the individual duration of the unemployment may be a risk factor for developing heavy drinking patterns [35]. Also, the relationship between substance use and unemployment is likely to be influenced not only by the duration of the unemployment but also by the overall employment rate for the area [22,36]. Indeed, becoming unemployed at a time of strong economic growth may have substantially different psychological and social implications than losing one’s job in times of recession [22]. Of importance within this context is that having a (pre-existing) history of mental health problems increases the vulnerability to suffer more severe consequences, *i.e.*, losing a job and becoming unemployed during economic crisis periods [37]. Indeed people suffering from mental illness are among the first to lose jobs during periods of economic downfall. Effects are especially harmful since having meaningful employment is one of the most important factors related to the sustained recovery of people with mental illness and/or substance use disorders. Overall, one of the consequences of the recession in Europe is that the gap in employment between individuals with and without mental health problems has widened significantly. These findings suggest that times of economic hardship may intensify social exclusion; in particular men and individuals with lower education seem to be affected most negatively [37].

Taken together, economic hardship undoubtedly increases the number and intensity of different sources of stress people have to deal with. Specifically subgroups that are vulnerable due to job-loss, unemployment or mental health problems experience a disproportional excess of stress. Independent of the type of stressor, an abundance of literature illustrates the intimate relationship between stress and a variety of substance use related consequences. Basically, stress seems to increase the risk of initiating substance use, of transitioning to regular (excessive) use, and ultimately the development of substance use disorders [38]. This may also explain that former addicts relapse back into their smoking or drug behavior during EC, as suggested by the findings of Gallus *et al.*, 2011 [16] and Kondilis *et al.*, 2013 [19]. Overall findings suggest that the effect of EC can compound existing socio-economic disadvantage and lead to an increase in societal inequity, including substance use effects.

Finally, we found remarkable differences between types of drugs of abuse. For alcohol and cigarette smoking, the economic crisis was associated with an overall decrease in the population, largely through an income effect. In contrast, the limited number of studies on drugs shows a consistent increase of use during the EC. It is interesting that these studies point to a change in the drug market, *i.e.*, more drug use, less use of expensive drugs like cocaine and an increase in cheaper laboratory made drugs such as amphetamines [20]. This is consistent with earlier U.S. studies showing that increases in poverty are associated with both an increase in illicit drugs use and an expansion of the illegal economy, *i.e.*, crime, sex work and drug dealing [22,33]. Indeed, in a U.S. study, Arkes *et al*. [39] provided evidence that a weaker economy led to greater teenage use of marijuana as well as hard-drug use. They found that teenagers were more likely to sell drugs in weaker economies, and suggested one mechanism for counter-cyclical drug use—that access to illicit drugs is easier when the economy is weaker. Apparently, in contrast with legal substances, the illicit drug market is much more flexible. The downward trend in the price of illicit drugs during the last decade in both the U.S. and E.U. can be interpreted as an adaption to the economic downturn [40]. In addition, compared to alcohol use the relationship between unemployment and increases in illicit drug use is much more consistent. Experiencing unemployment was associated with increased hazard of starting cannabis use in a U.S. study [41].

Finally, our findings on tobacco smoking suggest a dual pattern, *i.e.*, reduction of smoking for large parts of the populations and difficulties to quit in people who are disproportionally affected by the crisis (e.g., unemployment) [18]. The acute effects of losing a job have been earlier associated with relapse in smoking behavior within the U.S. [42]. These findings are consistent with a recent U.S. study, showing that the 2008 financial crisis had a weak effect on overall smoking prevalence. The pro-cyclical relationship (*i.e.*, the crisis results in a lower number of smokers) found among the employed is offset by the counter-cyclical relationship (*i.e.*, the crisis results in a higher number of smokers) found among unemployed individuals [43]. Importantly, within the U.S., teenagers and young adults increase cigarette use when the economy is weaker [44]. Thus, from this (US) context, public health interventions should specifically target both teenagers and those in unemployment, particularly in times of economic downfall. Whether this accounts for the European situation remains to be explored. Indeed, the scarce number of studies found within the current review and the fragmented information they offer, do not allow drawing, at this point, any conclusions. However, a recent Spanish study, published after the closing date of our review, showing unemployment in young (16–24 years) men associated with increased tobacco consumption, consistent with the U.S. findings [27,44].

Our review has several limitations. Foremost we need to acknowledge that the number of studies on this topic within the E.U. countries is very limited and methodology and sample choice varies widely. Although we think that the most relevant literature has been covered within our search the low number of studies found may be in part due to the fact we limited our search to Pubmed database. Overall, we need to acknowledge that the findings provide a fragmented information pattern that, although important and indicative in support of some hypotheses, warrants prudent interpretation. Next, we limited ourselves to traditional substances of abuse, leaving out important others, e.g., prescription drugs, legal highs and behavioral addiction. Although this is a limitation, we do think that alcohol, nicotine and to a lesser degree the more traditional illicit drugs account by far for the largest portion of burden due to substance use patterns in our society. In addition, many causal pathways may be involved that connect economic hardship experiences with different health behaviors [41]. Not many studies included in this review take this complexity into account; even when they do they may use only one measure or a limited number of variables, e.g., unemployment, leaving ample room for many confounding factors. Thus, findings need to be seen as indicative rather than conclusive. Finally, we limited the review to E.U. countries. Of importance, the E.U. is developing its own alcohol- and drug policies for the E.U. member states, so information based upon studies within these countries is highly relevant in this context. However this choice implicates that many other regions, e.g., U.S. and eastern European countries such as Russia were not included. These countries have often a different substance use culture so our findings need to be interpreted carefully when comparing with these countries.

## 5. Conclusions

Taken together, the results of the current overview illustrate that the effects of the economic crisis on changes in substance use patterns and their subsequent consequences are not unequivocal and are mediated by different factors. Identification of these factors may allow for identification of population subgroups, which are more vulnerable to the effects of economic hardship. Specifically, young men confronted with enduring longer standing unemployment may be at higher risk for substance use disorders and related problems during economic crises. Targeted interventions may help to reduce risks and alleviate potential negative consequences. In addition, and on a broader scale, interventions to combat economic exclusion and to promote social participation of individuals with mental health problems are key in times of economic crisis [37,45].

Finally, it is the strong opinion of the authors that interventions are not only important during, but even more so during the aftermath of economic crisis periods. Indeed, it is to be feared that during economic revival (as currently is happening in some parts of Europe though on a very modest scale) a substantial portion of the population may continue to suffer the (social) consequences of the crisis, being left behind and unable to benefit from economic growth. For these people continuing debt and unemployment can potentially make them especially vulnerable to developing new problems. This can in turn be a further barrier to alleviating existing mental health and substance use problems. These people are at risk of becoming a forgotten group, when the wave of attention for the effects of the economic crisis has waned (e.g., dramatic reduction of publication on the topic). So we, as professionals in mental health and substance use disorders, have an obligation to keep focused and continue to raise awareness, especially in the periods of economic recovery following any economic crisis.
